# QTc interval prolongation due to spinal anesthesia in patients with and without diabetes: an observational study

**DOI:** 10.1186/s12871-022-01614-8

**Published:** 2022-05-13

**Authors:** Jang-Ho Song, Chunwoo Yang, Woojoo Lee, Hongseok Kim, Youngjun Kim, Hyunzu Kim

**Affiliations:** 1 Department of Anesthesiology and Pain Medicine, Inha University College of Medicine, Incheon, Korea; 2 Department of Public Health Science, Graduate School of Public Health, Seoul National University, Seoul, Korea

**Keywords:** Corrected QT interval, Diabetes mellitus, Long QT syndrome, Spinal anesthesia, Subarachnoid blockade

## Abstract

**Background:**

Spinal anesthesia and autonomic neuropathy (caused by diabetes) prolong the QTc interval. Changes in the duration of the QTc interval following subarachnoid blockade in patients with diabetes have not been evaluated. We hypothesized that after subarachnoid blockade, QTc interval prolongation would be greater in patients with diabetes than in those without. Accordingly, we compared the QTc interval, T wave peak-to-end interval (Tp-e interval), blood pressure, heart rate, and heart rate variability before and after spinal anesthesia in patients with and without diabetes.

**Methods:**

This prospective observational study (Clinical Research Information Service identifier: KCT0004897) was conducted in a tertiary university hospital and included 24 patients with diabetes mellitus (DM group) and 24 patients without it (control group) who were scheduled for spinal anesthesia. The QTc interval, Tp-e interval, heart rate variability, blood pressure, and heart rate were measured before (T1) and 1 (T2), 5 (T3), and 10 min (T4) following subarachnoid blockade.

**Results:**

Ten minutes following subarachnoid blockade, the QTc intervals of patients in the DM group were significantly longer than the baseline values, whereas the change in the QTc interval in the control group was not significant (*p* < 0.0001 vs. *p* = 0.06).

**Conclusion:**

Spinal anesthesia caused a more significant prolongation of the QTc interval in patients with diabetes than in those without.

## Background

Diabetes mellitus (DM) causes worrying complications such as neuropathy, nephropathy, and retinopathy. Among these complications, autonomic neuropathy associated with the cardiovascular system could lead to a prolongation of the corrected QT (QTc) interval. QTc interval prolongation is associated with mortality risk regardless of the presence of autonomic neuropathy, and as such, can be considered a predictor of mortality risk in itself [1]. Patients with prolonged QTc intervals are prone to development of ventricular arrhythmias including unique *torsades de pointes,*and sudden cardiac death [2–5]. It is worth noting that cardiovascular complications are the most common causes of mortality and morbidity in patients with DM [6].

Neuraxial anesthesia is commonly used for lower-abdominal, pelvic and lower limb surgeries. Compared to general anesthesia, neuraxial anesthesia, including spinal anesthesia, has various advantages, such as a decreased risk of deep vein thrombosis, a lower likelihood of perioperative transfusion, and reduced rate of cancer recurrence [7–9]. The subarachnoid blockade (SAB) is a simple technique, which anesthesiologists are likely to be familiar with; however, it has multiple effects on the systemic circulation. Spinal anesthesia may cause a significant prolongation of the QTc interval; however, no specific mechanism for this phenomenon has been identified [10, 11].

Spinal anesthesia has several advantages over general anesthesia; for patients with DM, spinal anesthesia may be more effective than general anesthesia for the control of oxidative stress during lower limb amputation surgery [12]. In patients with DM, the QTc interval reportedly lengthens with the progression of DM neuropathy. It is necessary to evaluate the magnitude of the changes in QTc intervals that occur when spinal anesthesia is induced in patients with DM.

In this study, we hypothesized that QTc interval prolongation following spinal anesthesia induction would be greater in patients with DM than in those without. Therefore, we aimed to compare the values of the QTc interval, T wave peak-to-end (Tp-e) interval, blood pressure, heart rate, and heart rate variability before and after spinal anesthesia induction in patients with and without DM.

## Methods

The current prospective study was approved by the Institutional Review Board of Inha University Hospital, Incheon, Republic of Korea (Chairperson Prof. Gwang-Seoung Choi, N° IH2019-09–035, date of approval 25 November 2019). The study was registered at the Clinical Research Information Service (reference number KCT0004897, date of registration 26 November 2019, principal investigator H. Kim).

This study adheres to the STROBE guidelines and all study procedures followed the Declaration of Helsinki, 2013. Written informed consent was obtained from adults who were to undergo elective lower abdominal or lower limb surgery under spinal anesthesia. Twenty-four patients without DM (control group) and 24 patients with DM (DM group), aged 20–80 years (with American Society of Anesthesiologists Physical Status classifications ranging from ASA 1–3), were included in this study. Patients with DM were defined as those who took or injected themselves with antidiabetic medication and had a hemoglobin A1c (HbA1c) level ≥ 7.5%. We excluded patients who had a preoperative QTc interval > 440 ms, abnormal preoperative electrocardiogram (ECG) findings associated with conduction disorders or arrhythmia, a history of heart disease or circulatory insufficiency, or any preoperative electrolyte abnormality. In addition, patients who took medications known to affect the QTc interval were also excluded. Furthermore, patients with any contraindications to spinal anesthesia (increased intracranial pressure, infection at the injection site, coagulation disorder, severe hypovolemia, and aortic and/or mitral stenosis), BMI > 40 kg. (m^2^)^−1^, a history of spinal surgery, or congenital spinal deformity were excluded. The first and last participants were enrolled on 26 November 2019 and 5 February 2021, respectively. All data were collected between 8:00 AM and 11:30 AM to avoid the influence of the circadian rhythm on the QTc interval. All the patients with DM took their medications according to the recommendations of their endocrinologists and were not premedicated. After entering the operating room, the patients’ condition was monitored through electrocardiography, pulse oximetry, and a non-invasive measurement of blood pressure every 5 min. After the patients had received 5 ml.kg^−1^ of crystalloid preload over 15 min, continuous EEG lead II data were collected using the LabChart® data analysis software (version 8, ADInstruments, Colorado Springs, CO) and a data acquisition system (PowerLab®; ADInstruments). For heart rate variability (HRV) analysis, ECG acquisition was recorded for 10 min and precautions were taken to ensure that there were no external stimuli to the patients.

The patients were placed in the lateral decubitus position with their knees flexed against their abdomen or chest, assuming a fetal position. All procedures were performed by expert anesthesiologists with more than 3 years of experience. After local disinfection, a local anesthetic (2 ml of 2% lidocaine) was administered through injection at the L3–L4 interspace; spinal anesthesia was induced using a 27-gauge Quincke-type needle and a 22-gauge introducer. In all cases, the needle was inserted horizontally using the midline approach. After the subarachnoid space was identified through the outflow of cerebrospinal fluid, 0.5% 9–11 mg hyperbaric bupivacaine solution (Marcaine 0.5% Spinal Heavy®, AstraZeneca AB, Sodertalje, Sweden) was administered. Other additive drugs were not used for the augmentation of intensity or duration. Immediately following the injection of the drugs, the patient was placed in the supine position. The level of sensory blockade was assessed by the pinprick-sensation examination. No surgical procedure was performed during the 10 min that followed the return of the patient to the supine position, and ECG acquisition was repeated once.

The QTc interval, Tp-e interval, and hemodynamic-parameter were recorded before SAB (T1), and 1 min (T2), 5 min (T3), and 10 min (T4) after SAB.

The QT intervals were measured from the beginning of the QRS complex to the end of the T wave. Four sequential values were applied to Bazett’s formula (QTc = QT/√RR) to correct for the effect of the heart rate on the QT interval, and then averaged. The Tp-e interval from the peak of the P wave to the end of the T wave was measured automatically. For HRV analysis, frequency-domain HRV indices were obtained through power-spectral-density analysis using the fast Fourier transform. The values for two major power-spectrum components, high frequency (HF, 0.15–0.4 Hz) and low frequency (LF, 0.04–0.15 Hz), were obtained. The LF/HF ratio was also calculated.

The doses of hyperbaric bupivacaine, sensory blockade onset and recovery time, and levels of SAB used for both groups were recorded. Complications, including paresthesia, bloody taps, and post dural puncture headaches were also recorded.

Sample size was calculated based on a previous finding of a 20 ± 23 ms change in the QTc interval following SAB in a normal adult [11] and a normal QTc interval of 400 ± 25 ms in patients with DM [13]. To prove the hypothesis that the change in the QTc interval is greater than 40 ms for patients with DM, 21 patients were required for either group, based on α = 0.05. Assuming a loss-to-follow-up rate of 10% of included patients, we calculated that a total sample size of 48 patients was required to achieve a power of 80% for the study. Statistical analyses were performed using IBM® SPSS® Statistics 19.0 (SPSS Inc., IBM, Chicago, IL). All data were expressed as mean (standard deviation), number (%), or median [interquartile range], as indicated. After testing for data distribution normality using the Kolmogorov–Smirnov test and Shapiro–Wilk test, we analyzed continuous and categorical variables using a two-sample t-test and chi-squared test, respectively. The variables that did not show normal distribution were analyzed using the Mann–Whitney U test. For the comparison of differences in values of repeatedly measured variables between the two groups, Student’s t-test was used for each time point, and a general linear mixed model was used for the whole set of time points. Statistical significance was set at *p* < 0.05.

## Results

**Fig. 1:**
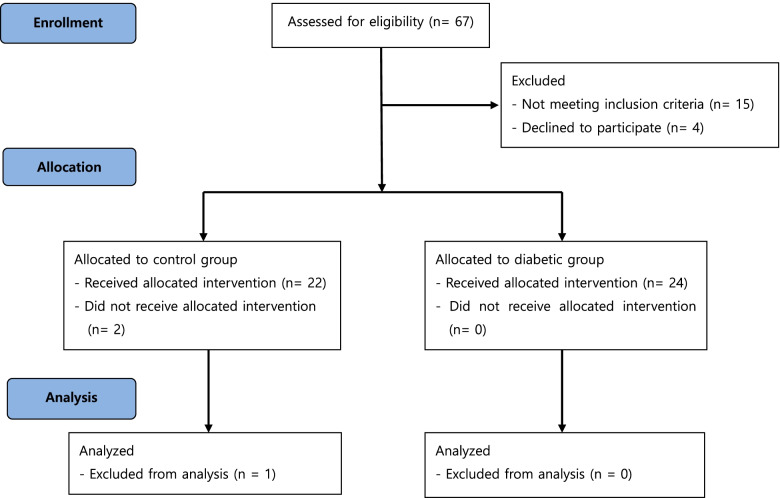
Flow diagram showing the flow of participants through each stage of our study

Ultimately, 21 and 24 patients in the control and DM groups completed the study, respectively. In the control group, one patient withdrew consent during the collection of ECG data; in another patient, SAB proved difficult, and consequently, general anesthesia was induced. Another patient was excluded during the final analysis because the pre-anesthetic QTc interval for that patient was over 440 ms. Figure 1 illustrates patient enrolment and the flow of the study. There were no significant differences in demographic and blockade-procedure characteristics between the two groups (Table 1). Five patients in the DM group were diagnosed with DM neuropathy. Patients in the DM group used diverse medications. Moreover, we could not establish the association between DM medication and the primary outcome that was QTc prolongation.

**Table 1:** Demographic and procedural data for the control and DM groups

	Control group (*n* = 21)	DM group (*n* = 24)	*p-*value
Age; years	61.3 ± 12.4	61.1 ± 10.5	0.943
Sex; Male/Female	15/6	19/5	0.801
Height; cm	165.3 ± 9.2	166.7 ± 7.9	0.592
Weight; kg	67.4 ± 12.5	67.7 ± 14.7	0.939
BMI; kg/m^2^	24.5 ± 3.1	24.4 ± 4.9	0.919
Length of DM; years	NC	10 (11)	-
HbA1c; mg/dl	NC	8.5 (2.7)	-
DM type; I/II	NC	1/23	-
End organ damage; 0/1/2	NC	18/5/1	-
H-M dosage^a^; mg	10.1 ± 0.8	10.2 ± 0.8	0.614
Onset time; min	7.8 ± 1.9	7.0 ± 1.3	0.14
Recovery time; min	116.2 ± 22.9	119.2 ± 19.5	0.644
Sensory block level	T11 (4)	T10 (2)	0.096

*DM* diabetes mellitus, *BMI* body mass index, *HbA1c* Hemoglobin A1c, NC

Values are expressed as number, mean ± standard deviation, or median [interquartile range]

^a^H-M

**Fig. 2:**
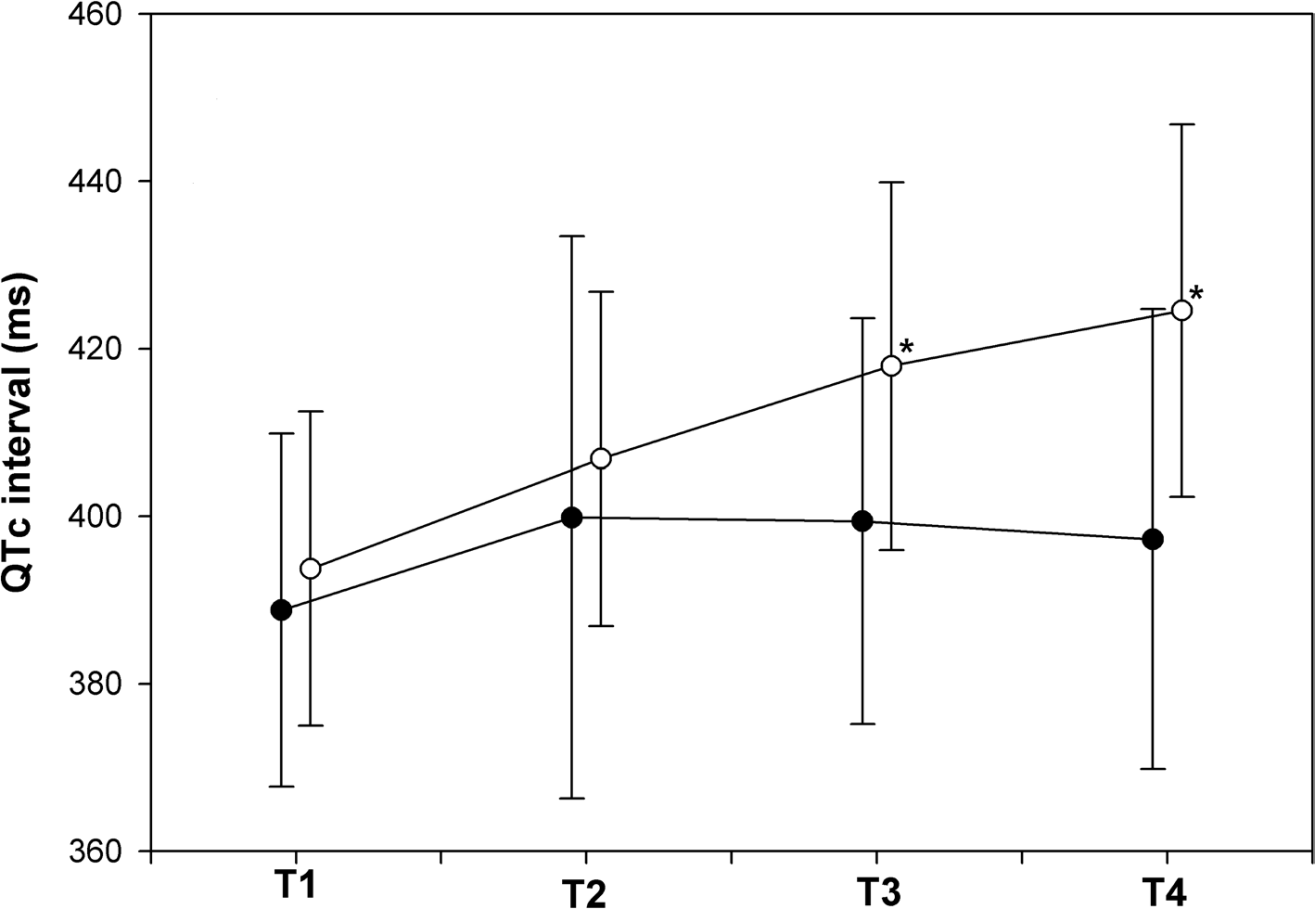
Comparison between changes in QTc intervals in patients with and without diabetes. Time 1, before spinal-anesthesia induction; time 2, 1 min following spinal anesthesia induction; time 3, 5 min following spinal anesthesia induction; time 4, 10 min following spinal anesthesia induction. Control group, ●; diabetic group, ○. * *p* < 0.05. QTc, corrected QT

The primary outcome in this study was the changes in the QTc interval from before administering SAB to 10 min after administering SAB. The changes in QTc were 8.5 ± 19.9 ms and 30.8 ± 17.5 ms in the control and DM groups, respectively, (*p* = 0.001). Fig. 2 and Table 2 show the changes in the QTc interval between both groups across time points. For patients in the DM group, the QTc interval was significantly longer 10 min following SAB than at baseline, while in the control group, the change in the QTc interval was not significant (*p* < 0.0001 vs *p* = 0.06).

**Table 2:** The QTc interval across different timepoints in the control and DM groups

		T1	T2	T3	T4	*p*-value (T1-T4)
QTc (ms)	C	388.8 ± 21.1	399.9 ± 33.6	399.4 ± 24.2	397.3 ± 27.4	0.06
	DM	393.8 ± 18.8	406.8 ± 19.9	417.9 ± 22.0	424.5 ± 22.2	0.0001
		0.378	0.919	0.012	0.002	0.003

The OTc intervals were recorded before subarachnoid blockade (SAB) (T1), and 1 min (T2), 5 min (T3), and 10 min (T4) following SAB

*ΔQT*_*C*_: change in corrected QT, *DM* diabetes mellitus

Values are expressed as mean ± standard deviation

Additionally, the changes in the QTc interval from before to 10 min after SAB in the DM and control groups, which were divided according to international recommendations, [14] were summarized in Table 3 and 4. The prevalence of the absence of changes in the QTc interval and moderate, marked, and substantial changes in the QTc interval varied between groups (*p* = 0.013). Four (17%) and two (8%) patients in the DM group demonstrated a QTc interval of 450–480 ms and 481–500 ms at 10 min, respectively, following SAB. On the contrary, one patient (5%) in the control group displayed a QTc interval of 450–480 ms, and this difference was statistically significant (*p* = 0.035).

**Table 3:** Prevalence of absent, moderate, marked, and substantial ΔQT_C_ in the control and DM groups

	Control (*n* = 21)	Diabetic (*n* = 24)	*p-*value
ΔQTc			
Absent	6 (29%)	0	0.0132
Moderate	12 (57%)	15 (63%)	
Marked	3 (14%)	7 (29%)	
Substantial	0	2 (8%)	

*ΔQT*_*C*_ change in corrected QT, *DM* diabetes mellitus

The prevalence of absent (ΔQT_C_ ≤ 0 ms), moderate (0 < ΔQT_C_ ≤ 30 ms), marked (30 < ΔQT_C_ ≤ 60 ms), and substantial (ΔQT_C_ ≥ 60 ms) values is indicated by the number (proportion) of patients

**Table 4:** Electrocardiographic and hemodynamic parameters at four different time points for patients in whom spinal anesthesia was induced

	T1	T2	T3	T4
Tp-e interval; ms				
Control	65.9 ± 15.3	67.6 ± 12.9	70.9 ± 16.3	67.1 ± 11.4
Diabetic	61.5 ± 11.5	60.7 ± 10.7	68.3 ± 12.2	69.0 ± 13.0
Mean blood pressure; mmHg				
Control	105.1 ± 13.4	103.2 ± 13.6	94.3 ± 13.7	96.0 ± 13.8
Diabetic	106.8 ± 18.2	105.5 ± 20.1	96.1 ± 18.0	98.6 ± 20.4
Heart Rate; bpm				
Control	69.9 ± 7.9	70.2 ± 11.8	72.2 ± 12.5	71.9 ± 12.0
Diabetic	77.0 ± 14.3*	80.3 ± 14.5*	80.3 ± 14.4*	80.5 ± 14.1*

Tp-e interval and hemodynamic-parameter values were recorded before subarachnoid blockade (SAB) (T1), and 1 min (T2), 5 min (T3), and 10 min (T4) following SAB

Values are expressed as mean ± standard deviation

*SAB* subarachnoid blockade, *Tp-e interval* T wave peak-to-end interval

There were no significant differences between the groups with respect to the values for the Tp-e interval and systolic, diastolic, and mean blood pressure at each time point or in comparison to baseline values. The heart rates were significantly higher in the DM group than in the control group throughout all time points; these data are summarized in Table 3 and 4, while the changes in the HRV indices in the DM and control group are summarized in Table 5. In both groups, before and after SAB, there was no significant difference in total power, HF, LF, and the LF/HF ratio. No patient exhibited arrythmias on ECG monitoring for 3 h following spinal anesthesia. No other adverse events occurred in either group.

**Table 5:** Power-spectral heart rate variability data

	Total power; ms^2^		High frequency (HF); ms^2^		Low frequency (LF); ms^2^		LF/HF ratio	
	Before	After	Before	After	Before	After	Before	After
Control	794 (1878)	1039 (1587)	198 (510)	183 (216)	209 (504)	189 (418)	1.2 ± 1.6	1.3 ± 1.2
Diabetic	800 (2306)	729 (1885)	165 (654)	103 (480)	216 (486)	112 (480)	1.5 ± 1.7	1.7 ± 1

Values are reported as mean ± standard deviation or median [interquartile range]

## Discussion

In this prospective study, we compared the changes in the QTc interval before and then at 1, 5, and 10 min after SAB in patients with and without DM. When the baseline QTc interval or the QTc interval of patients without DM was compared to those of patients with DM, we found it to be significantly prolonged at 5 and 10 min following SAB.

To the best of our knowledge, this is the first study that compared the QTc interval prolongation following SAB of patients with and without DM. The major finding was that the QTc interval prolongation following SAB was greater in the patients with DM than in the controls. The prevalence of significant changes in the QTc interval from pre-SAB to 10 min after SAB in the DM group was 29% and 8%, respectively, whereas in the control group, the corresponding values were 14% and 0%, respectively. In this study, the incidence of QTc interval > 450 ms was 25% in the DM group. One-third of the patients in the DM group experienced a QTc interval prolongation of > 30 ms. In a previous study, the change in the QTc interval was 22 ms in patients in whom spinal anesthesia was induced [15]. In the present study, the results show that the change in the QTc interval after SAB was 31 ms for the DM group and 11 ms for the control group. We speculated that the reason behind this difference in the results could be, like in the study by Duma et al., that data of participants with various conditions that cause QTc interval prolongation and of those who received medications that cause QTc interval prolongation were also included. Consequently, in that study, the identified changes in the QTc interval were greater than the changes identified in the present study.

Many anesthesiologists prefer using regional blockade for patients with long QT syndrome since QTc interval prolongation is greater following general anesthesia induction than after spinal anesthesia induction [16, 17]. The change in the QTc interval in patients under general anesthesia was 33 ms, while the change in patients under spinal anesthesia was 22 ms [15]. Although this QTc interval prolongation after SAB may be considered insubstantial, it should be noted that we enrolled patients with DM who had normal QTc intervals under 440 ms. If the patients with DM already had prolonged QTc intervals, a 30-ms QTc interval prolongation could be dangerous. According to the findings of a previous study, there is a direct correlation between the QTc interval and the progression of diabetic autonomic neuropathy [13]. In the present study, due to not including patients with DM who had severe neuropathy, the results showed that the baseline QTc interval in the DM group was below 400 ms and comparable to that of the control group.

Therefore, the risk of QTc interval prolongation should not be overlooked in all patients with DM. The QTc interval should be carefully monitored throughout the perioperative anesthesia period in patients with DM receiving spinal anesthesia. Importantly, for patients with DM who already have prolonged QTc intervals, strategies for the attenuation of the activation of sympathetic outflow should be considered.

The specific mechanism underlying QTc prolongation following SAB can be explained by an imbalance between lumbar and thoracic sympathetic activity following spinal anesthesia. In spinal anesthesia, it is difficult to directly affect the sympathetic fiber in T1-T4. However, SAB in the lumbar or lower thoracic level stimulates thoracic sympathetic outflow above the blockade, thus causing reflex sympathetic activation [18]. Subsequently, myocardial repolarization increases particularly through the cardioaccelerator nerve fibers and indirectly prolongs the QTc interval [19]. Sympathetic fibers are more sensitive than sensory or motor neurons; thus, the aforementioned reflex and QTc prolongation occur immediately rather than after a somatosensory block [20]. In addition, hemodynamic changes, such as hypotension, trigger sympathetic activation. Hypotension stimulates the baroreceptors that accelerate the activation of sympathetic outflow [15].

We investigated HRV following SAB to reveal the relationship between a change in the autonomic nervous system and SAB. Unfortunately, our HRV data did not indicate that there was any relevant difference either between the two groups or before and after SAB. A previous study found that SAB reduced both the LF and HF components of HRV, and this finding coincided with the cephalad spread of the blockade. The sensory block level reached above T5–6 in all patients, and the change in HRV amplitude was more evident than that in our study. Especially, the power spectra were almost abolished in patients reaching the sensory blockade level of T1–2 [20]. On the other hand, the HRV changes in our study were not statistically relevant since the median value of the sensory blockade level in this study was T10–11.

According to previous measurements of QTc intervals after SAB, QTc interval prolongation is greatest 10 min after blocking and decreases thereafter [11]. In this study, we measured the QTc interval until 10 min after SAB. As shown in Fig. 2, the change in the QTc interval in the control group was not significant 1 min after SAB but increased and decreased slowly. On the contrary, the QTc interval was significantly prolonged in the DM group at 5 min and 10 min following SAB. Moreover, it was likely to maintain the up-slope, which was significantly longer than that at baseline. The prolongation of the QTc interval following spinal anesthesia is maintained until post-surgery [15], consistent with our results. In other words, QTc prolongation following spinal anesthesia depends on the patient characteristics. In future studies, researchers should measure the QTc interval until the recovery of the effect of SAB or for 12 h, considering the difficulty in identifying the time of recovery to a baseline value.

The Tp-e interval is a reliable predictor of the risk of *torsades de pointes*because the Tp-e-interval value is a surface ECG marker of the transmural dispersion of repolarization across the myocardial wall [21, 22]. In this study, we found that the Tp-e interval did not increase significantly following SAB either in the DM or in the control group. This result concurs with that of a previous study of ECG changes after SAB [11, 23]. This finding supports the rationale that spinal anesthesia itself is relatively safe for patients with DM who have normal QTc intervals, assuming there is no other insult to increase the QTc interval. It is unknown what the results will be with respect to patients with both DM and QTc interval prolongation. The monitoring of QTc and Tp-e intervals is necessary in cases where spinal anesthesia is required for patients with DM who also have severe QTc interval prolongation.

Considering the hemodynamic changes, blood pressure values did not differ significantly between groups. Hypotensive events could affect the results of the QTc interval; thus, sufficient hydration was delivered to all participants before SAB and the doses of the anesthetic agent were carefully titrated. The autonomic function of patients with DM undergoing spinal anesthesia was relatively maintained, whereas those undergoing general anesthesia could supposedly experience profound hypotension owing to cardia autonomic neuropathy [24].

The heart rates were higher in the DM group than in the control group. This difference was maintained throughout all time points, regardless of the induction of spinal anesthesia. Previous studies have shown that this increase in the heart rate in patients with DM is due to autonomic neuropathy [25, 26]. Notably, in old age, a higher heart rate may be associated with undiagnosed DM and mortality [27]. The majority of anesthesiologists usually focus on blood pressure during spinal anesthesia; nonetheless, increased heart rate can be a clue to hemodynamic changes in patients with diabetes.

There were certain limitations to this study. First, five patients in the DM group were diagnosed with diabetic neuropathy, and it was not clear whether they had progressed to autonomic neuropathy. It is difficult to predict the extent of QTc interval prolongation in patients with both DM and severe neuropathy. Second, the QTc interval was only measured for up to 10 min after SAB; this may be a short period to evaluate its clinical impact and determine longer lasting implications. However, the duration of 10 min was chosen to avoid any delays in the surgery. Third, the variables considered in the present study affect the type and dosage of the administered local anesthetics and the blockade level. Thus, the results can vary if aspects of SAB change.

## Conclusion

In conclusion, QTc interval prolongation due to spinal anesthesia was found to be significantly greater among the patients with DM than in those without.

## Data Availability

The data are not available for public access because of patient privacy concerns but are available from the corresponding author on reasonable request.

## Abbreviations

HF: High frequency
HRV: Heart rate variability
LF: Low frequency
